# Improving species distribution models of zoonotic marine parasites

**DOI:** 10.1038/s41598-019-46127-6

**Published:** 2019-07-08

**Authors:** Katharina G. Alt, Judith Kochmann, Sven Klimpel, Sarah Cunze

**Affiliations:** Goethe-University, Institute for Ecology, Evolution and Diversity; Senckenberg Biodiversity and Climate Research Centre, Senckenberg Gesellschaft für Naturforschung; Max-von-Laue-Str. 13, D-60438 Frankfurt/Main, Germany

**Keywords:** Ecological modelling, Biogeography

## Abstract

Environmental niche modelling is an acclaimed method for estimating species’ present or future distributions. However, in marine environments the assembly of representative data from reliable and unbiased occurrences is challenging. Here, we aimed to model the environmental niche and distribution of marine, parasitic nematodes from the *Pseudoterranova decipiens* complex using the software Maxent. The distribution of these potentially zoonotic species is of interest, because they infect the muscle tissue of host species targeted by fisheries. To achieve the best possible model, we used two different approaches. The land distance (LD) model was based on abiotic data, whereas the definitive host distance (DHD) model included species-specific biotic data. To assess whether DHD is a suitable descriptor for *Pseudoterranova* spp., the niches of the parasites and their respective definitive hosts were analysed using ecospat. The performance of LD and DHD was compared based on the variables’ contribution to the model. The DHD-model clearly outperformed the LD-model. While the LD-model gave an estimate of the parasites’ niches, it only showed the potential distribution. The DHD-model produced an estimate of the species’ realised distribution and indicated that biotic variables can help to improve the modelling of data-poor, marine species.

## Introduction

Ecological niche modelling (ENM) is a popular tool to examine the ecological and spatial limitations of species^1,2^. Thus, the availability of representative occurrence data is crucial for good modelling outcomes^3^. In times of global change during which species are facing climate shifts and habitat loss, models help identify suitable habitats for species and project potential distributions of species in the future^4–6^. The motivation of modelling a species’ niche can be diverse, from conservation approaches of endangered species^7–9^ to the risk assessments of disease vectors and zoonotic agents^10–13^.

While niche modelling is widely applied and well established for terrestrial species^14–17^, aquatic, especially marine organisms pose additional challenges. Data is not only rarer because marine species are hard to monitor, but also the marine environment itself is less well studied, because we can only observe a small fragment of it. Thus, for marine species it may be even more difficult to obtain good and meaningful occurrence data than for terrestrial species^18^. As a result, there are much fewer studies using ENM for marine species compared to terrestrial species (e.g.^19–27^). In particular, there are only few studies focussing on the distribution of marine endoparasites^28^. Marine endoparasites are harder to observe than other marine organisms, because they are concealed within their host. Their complex life cycles result in a degree of dependency on other organisms. It seems therefore unlikely to describe the niche of a parasite in a meaningful way without taking into account biotic interactions.

Most parasites are only host-specific regarding their definitive host, but not regarding intermediate hosts, which means that intermediate host interactions are less suitable descriptors. If the definitive host is a marine mammal, data acquisition of parasites is problematic, because of the boundaries of non-invasive detection methods. However, including parasite occurrence data from intermediate hosts (especially fishes) and definitive host distribution data separately could improve the ENM of marine parasites.

To evaluate the applicability of this concept, we chose the ascaridoid nematode species complex *Pseudoterranova decipiens* (*s.l*.). The so-called codworm is a parasite of commercially relevant gadid fish species that can be found in the shelf regions throughout almost all latitudes. Apart from high infection levels in their teleost hosts that lead to a contamination of the fish filet and thus, a decrease in value, *Pseudoterranova* spp. are known as cause of the zoonotic disease Anisakidosis^29–32^. Hence, knowledge of the distribution of these parasites is of interest to identify fishing areas at risk for a high parasite load in the respective food fish.

Previously described as a single species, *Pseudoterranova decipiens* (*s.l*.) has become recognised as a complex of species. The genus now includes species from different regions, *P. azarasi* Yamaguti & Arima, 1942; n. comb. Mattiucci *et al*., 1998, *P. bulbosa* Mattiucci *et al*., 1998, *P. cattani* George-Nascimento & Urrutia, 2000, *P. decipiens* (*s.s*.) and *P. krabbei* PAGGI *et al*.,2000^33–36^. Moreover, *P. decipiens* E has been described as a species candidate from the Southern Ocean^37,38^. All these species have different geographical distributions (which could suggest different climatic tolerances) and definitive hosts, which means they cannot be modelled together as a species complex.

After a benthic larval stage during which the larvae are attached to bottom substrate the life cycle of the genus *Pseudoterranova* includes three hosts. Like all nematodes of the family Anisakidae, *Pseudoterranova* spp. require a first crustacean host (copepod) and optionally other macroinvertebrates, a second teleost (or squid) host, and a marine mammal as definitive host, more precisely Pinnipedia^39^. The ecology of seals involves the formation of terrestrial colonies (some species also use pack ice) during the mating season, which are used as haul out sites^40^. In consequence, the concentration of the parasites’ eggs should be high in areas where the definitive hosts aggregate^41–45^. A connection between the presence of pinniped colonies and the codworm burden in fish has been made ever since protective measures following commercial seal hunt led to the recovery of seal populations^46^. This makes definitive host occurrences a potentially well-suited indicator for codworm distribution^41,46,47^, which can be used in modelling the parasites’ ecological niches. A benefit of this approach is that most seals are much better monitored and recorded than other marine mammals that do not have terrestrial haul out sites.

Based on this knowledge we aim at comparing the niches of potentially zoonotic marine parasites from the *Pseudoterranova decipiens* complex with those of their definitive hosts, in both, geographical and ecological space. The overall aim was to reveal whether ecological niche models of marine parasites can benefit from including definitive hosts, thus, better project distributional patterns of marine parasite species than those models including only abiotic variables.

## Results

**Figure 1:**
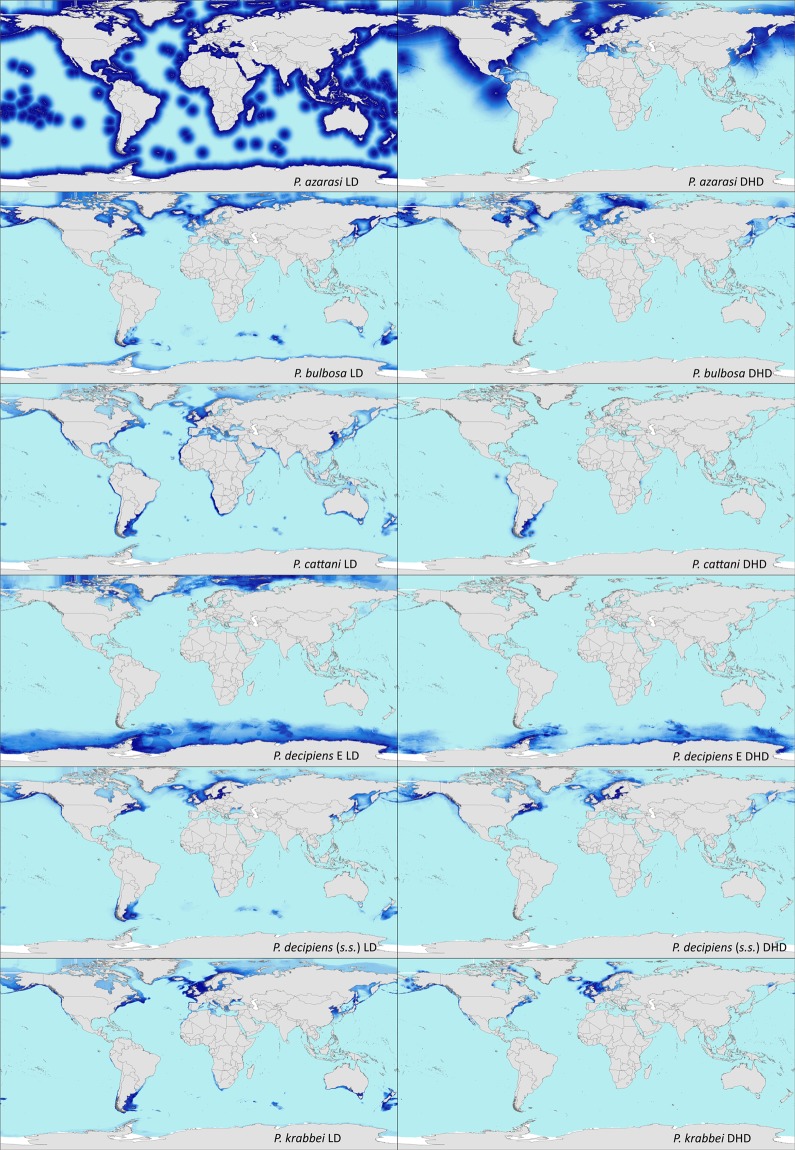
Modelled distribution of *Pseudoterranova* spp. Habitat suitability maps (WGS-84 projection) of *Pseudoterranova azarasi, P. bulbosa, P. cattani*, *P. decipiens* E*, P. decipiens* (*s.s*.) and *P. krabbei*, using the LD-model and the DHD-model. The modelled habitat suitability proportionally increases with colour intensity.

Georeferenced, molecularly identified occurrences of *Pseudoterranova decipiens* (*s.s*.) (n = 46), *P. bulbosa* (n = 31), *P. cattani* (n = 15), *P. krabbei* (n = 8), *P. azarasi* (n = 5) and *P. decipiens* E (n = 4) were compiled from published literature (Table S1)^30,32,33,35–37,48–75^. Parasite and host occurrence data were plotted on maps to visualise their distribution (Fig. S1, S2). The results are depicted in Fig. 1.

### Species distribution model improvement

The mapped habitat suitability for the six *Pseudoterranova* species, based on the land distance model (LD-model), did not differentiate between the northern and southern hemisphere. The LD-model for *P. decipiens* (*s.s*.) showed the highest habitat suitability in the temperate regions of the northern hemisphere, increasing towards the coast. Regions with high habitat suitability were in the Baltic Sea and the North Sea. Other modelled habitats were mapped in the North Atlantic, the North Pacific, along the coast of the Gulf of Alaska, the Sea of Okhotsk and in the Sea of Japan. Despite the sampling points being only from the northern hemisphere, modelled habitat suitability was also high around the coast off Patagonia and the Falkland Islands. The modelled habitat suitability of *P. bulbosa* was similar to *P. decipiens* (*s.s*.) but shifted towards the poles, into colder regions.

*Pseudoterranova cattani* had an austral distribution with high modelled habitat suitability in South America, especially in coastal regions off Peru, Chile, Argentina and the Rio de la Plata area. In the East Atlantic, the Benguela and the Canary Current region a very high habitat suitability was modelled. Furthermore, a high habitat suitability was found in the North Sea, Chinese Sea and the Atlantic and Pacific coasts off North America.

A high habitat suitability for *P. krabbei* was detected in the North Sea, the North East Atlantic and the North West Atlantic. In the Pacific, high habitat suitability was detected at the coast off Alaska and in the Sea of Japan west off Hokkaido and in the northern Chinese Sea. The suitable habitats in the southern hemisphere were off the Chilean and Argentinean coasts and the waters of the Bass Strait between Australia and Tasmania.

The habitat suitability for *P. azarasi* based on the LD-model was not able to provide any biogeographical information. High habitat suitability was modelled for *P. azarasi* in coastal regions in all geographical latitudes. This was unaffected by other environmental factors.

The modelled habitat suitability for *P. decipiens* E was highest in the polar regions. It covered the Southern Ocean with the habitat suitability increasing towards the land mass. A high habitat suitability was detected east of the Antarctic Peninsula, which is also the area where the samples originated from. In the northern hemisphere, the LD-model predicted a high habitat suitability in the Arctic Sea and the Barents Sea.

The habitat suitability models of the parasites gained accuracy through the introduction of definitive host distance (DHD), as indicated by the increase of area under the curve (AUC) values (Table 1). The AUC-values of the LD-model are > 0.9 for all *Pseudoterranova* species. In the DHD-model, AUC-values significantly increased (p < 0.05, Wilcoxon test). Maxent response curves of DHD showed the same effect for all analysed parasite species: with increasing DHD modelled probability of parasite presence monotonously decreased (Fig. S3).

**Table 1:** Area under the curve (AUC) values for the different modelling approaches.

Species	n	LD AUC	DHD AUC
*P. azarasi*	5	0.912	0.950
*P. bulbosa*	31	0.973	0.985
*P. cattani*	15	0.991	0.998
*P. decipiens E*	4	0.984	0.995
*P. decipiens* (*s.s*.)	46	0.980	0.987
*P. krabbei*	8	0.995	0.998

AUC-values of the Maxent LD-model and DHD-model of *Pseudoterranova azarasi*, *P. bulbosa*, *P. cattani*, *P. decipiens* E, *P. decipiens* (*s.s*.) and *P. krabbei*.

The DHD-model maps of all *Pseudoterranova* species seem to be more geographically accurate than the maps based on the LD-model, reflecting the observed realised distribution of the parasites (Fig. S1). In particular, the representation in the geographical space is changed, the hemisphere in which the species and its hosts do not occur is not included, despite its apt abiotic conditions. For example, the DHD-model for *P. bulbosa* only includes suitable habitats in the North Atlantic and North Pacific. This corresponds to where the specimens of *P. bulbosa* have actually been sampled (Fig. S1). Furthermore, the DHD-model reduces the habitat suitability projection from the whole North-Russian coast to the Barents Sea.

The variable contribution of the LD-model was incoherent between the six different *Pseudoterranova* species (Table S2). The DHD-model had a clear pattern, which can be attributed to DHD becoming the most important variable of the model. The Maxent response curves for all modelled parasites show that the habitat suitability monotonously decreased with increasing distance to a definitive host (Fig. S3).

The information of DHD contributed to all modelled species (Table S2), which makes this model superior to the LD-model which lacks this information entirely. This also applies to the species with the fewest occurrences, *P. decipiens* E, which relied almost exclusively on SST in the LD-model. Through the introduction of DHD, all other variables gain contribution (Table S2).

### Principal components analysis and niche plots

To compare the niches of the parasites and their respective definitive hosts, a PCA_env was calculated for the *Pseudoterranova* species with most occurrences, *P. bulbosa*, *P. cattani* and *P. decipiens* (*s.s*.). In this analysis the environmental variables of the LD-model (depth, land distance, mean sea surface temperature, primary production, salinity) were included.

**Figure 2:**
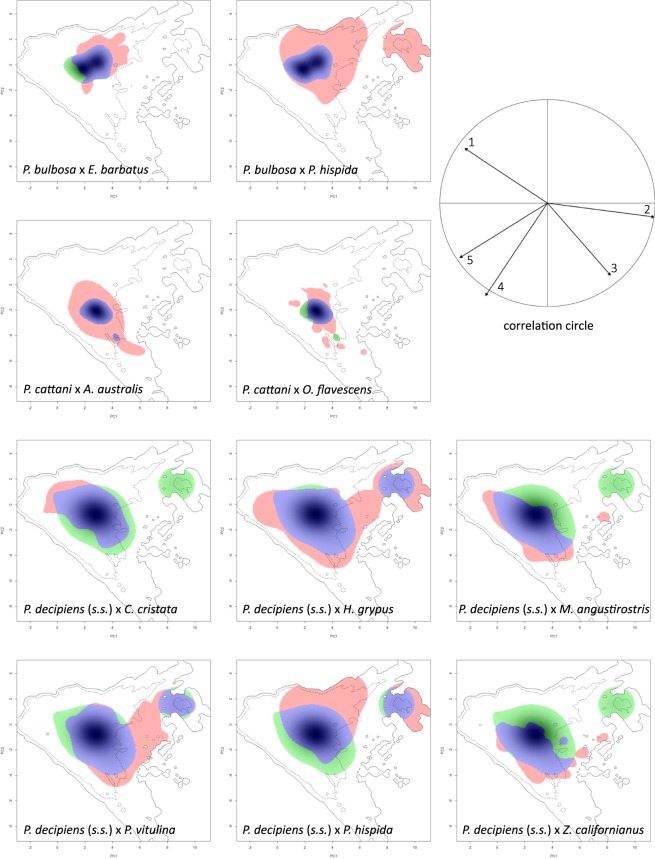
Parasite and definitive host niches in niche space. Niche plots of *Pseudoterranova bulbosa, P. cattani* and *P. decipiens* (*s.s*.) and their respective hosts in niche space, defined through principal components PC1 (x-axis) and PC2 (y-axis). Correlation circle of the variables used to calculate the principal components (axis 1 = 41.64%, axis 2 = 26.3%). Blue = niche overlap, green = parasite/unfilling, red = host/expansion, 1 = LD, 2 = Depth, 3 = Primary Production, 4 = mean Sea Surface Temperature, 5 = Salinity.

Schoener’s D was calculated as a measure of niche overlap. This revealed a high overlap of the niches of *P. bulbosa* and its definitive host *Erignathus barbatus* (D = 0.543), but a much lower overlap (D = 0.018) with the host *Pusa hispida* (Table S3, Fig. 2).

The niche plots generated in ecospat reveal the proportion of the parasite niche that is covered by the host niche, indicated by niche overlap between the parasite and its host (Fig. 2). Niche unfilling (green) indicates the proportion of the parasite niche that is not explained by the niche of the respective host. The niche of the parasite *Pseudoterranova bulbosa* is entirely covered by the niche of the ringed seal (*Pusa hispida*), resulting in no unfilling. The same applies to *P. cattani* and the South American fur seal (*Arctocephalus australis*). The niche of *P. decipiens* (*s.s*.) is modelled broader than the niches of the other parasite species. The host with the largest niche overlap is the grey seal (*Halichoerus grypus*), with the lowest unfilling of the parasite niche. The niche plots visualise that the definitive hosts cover different regions of the niche of *P. decipiens* (*s.s*.), resulting in a full niche cover of the parasite by a combination of its definitive host niches.

## Discussion

The approach undertaken here was based on the assumption that reproduction is the most basic requirement to sustain a population. In case of endohelminths, the reproduction depends on the availability of a definitive host, which enables the parasite to mature, mate and release eggs into the environment^39^. Hence, we regard the presence of definitive hosts as a suitable descriptor to help refine the models of *Pseudoterranova* species. We analysed whether including specific host presence records would improve the species distribution models of the parasites.

The modelling approach in Maxent was based on environmental variables available in the database GMED. The outcome for the different species varied depending on the occurrence sample size and quality. Most inaccuracies occurred for the species *Pseudoterranova azarasi*, which had the fewest and most inconsistent occurrence data, and would normally be considered unfit for modelling. As a result of data deficiency, the occurrences of this parasite could not be linked to all environmental variables in a meaningful way, hence, the model was exclusively based on land distance, with no other differentiation. But even the models of species with more occurrences were geographically inaccurate, because habitats which are out of the range of a species (by distance or continental barriers) were considered as suitable. Even if the model projected a high habitat suitability for a species from the North Atlantic in the southern hemisphere, the requirements of an endoparasite with a complex multi-host life cycle are likely not fulfilled. For non-parasitic species, the prediction of the LD-model might be sufficient, with mobility as the single limiting factor, preventing the species to colonise the modelled habitat^3^. But even then, biotic interactions and competition for resources might be further limiting factors. Since the life cycles of *Pseudoterranova* spp. are dependent on definitive host availability, potential niches need to include the host, as performed in an approach published by Kuhn *et al*.^28^. While the LD-model might give reasonable information on the potential distribution of a parasite, the DHD-model shows its realised distribution.

If a model were able to represent the fundamental niche of an organism (sensu Hutchinson^76^), its mapped habitat suitability would represent all potential habitats disregarding constraints by biotic interactions and mobility restrictions^77–79^. However, identifying the fundamental niche would only be possible under ideal circumstances, as it is combined from an organism’s realised response, ecological response and physiological response to its environment^80,81^. Species distribution models based on distributional data result in a representation of the species’ realised niche. To describe the ecological niches of the *Pseudoterranova* species, we need to acknowledge the life cycle of the parasite by using an approach accounting for the species’ biotic requirements. Georeferenced samples were taken from intermediate hosts, so these data are not necessarily useful in defining the niche of the hatched, free-living larval stage (which could be argued as the “abiotic” niche of the parasite). Working with occurrences from intermediate host stages is not ideal. However, if the model includes the definitive host data, by using DHD, it considers the paramount factor, for the parasite to reproduce. Hence, the resulting model should be a representation of the species’ realised geographical distribution.

The DHD-model considers the circumstance that the presence of host colonies has an impact on the concentration of larvae attached to the sea floor^39^. A study by McConnell, Marcogliese & Stacey^82^ investigated the sedimentation rate of *Pseudoterranova* spp. eggs. The benthic life cycle of *Pseudoterranova* requires the eggs to reach the seabed, which means their density needs to be higher than sea water. Structural factors like depth (which usually increases with increasing land distance) and currents are crucial factors for the sedimentation rate, while processes like upwelling could be an inhibitor. A high density of eggs and hatched larvae at the sea floor influences the prevalence of the parasite in its invertebrate intermediate host, and thus in the teleost host, where it is noticed because it decreases the commercial value of a fish and its relevance regarding food safety.

Seal hunting depleted the number of seals worldwide to 33 extant species. After hunting was widely abolished, regular monitoring has been conducted within the scope of conservation efforts. As seals are semi-terrestrial, monitoring them is much easier than other marine mammals. Therefore, seals are well monitored and there are many reliable presence records in databases like GBIF. A considerable advantage of the DHD-model is the data foundation, because the definitive hosts are much easier monitored than the parasites. Due to this advantage concerning the definitive host data, this study differs from the approach suggested by Kuhn *et al*.^28^, who used modelled definitive host habitat suitability as a predictor for the nematode genus *Anisakis*. Compared to pinnipeds, the occurrence data for some long-distance migrating cetaceans tend to be much more biased and are more difficult to acquire in the first place. In our case, the life cycle and ecology of *Pseudoterranova* allows us to use DHD. This approach removes the level of uncertainty created by using modelled host distributions, which might however be inevitable in other studies (e.g.^27,28^). We chose to favour DHD as a direct variable over modelling definitive host distribution, to reduce geographical misrepresentation and to avoid an additional modelling step, which would create an additional level of uncertainty. Using DHD provides a species-specific descriptor, which adds spatial information to the model. This spatial component may not be relevant to the parasite’s ecological requirements, but it is highly relevant for providing a realistic representation of the realised, geographical distribution. Using a modelled definitive host descriptor would not have included this kind of spatial information.

The difference in variable contribution between the LD-model and the DHD-model highlights the explanatory power of the DHD variable in lieu of land distance. While land distance had the highest variable contribution to the models of two species (*P. azarasi* and *P. cattani*), DHD was the most important descriptor of all analysed species except *P. decipiens* E, where it, together with SST, still contributed equally to the model. The quality of a single model cannot be assessed by its AUC-value, because it might be increased by a uniform (small) set of data. However, the AUC-value can be used to compare the effect of new descriptors on the same distributional data^83^. The increased AUC-values of all species models clearly show that the model was improved by the use of DHD instead of LD.

Due to the parasite specimens originating from intermediate instead of definitive hosts, we needed to show that a definitive host can occur at each parasite’s sampling site. If this is the case, there should be a clear overlap between the niches of the parasite and its associated definitive hosts. To evaluate the eligibility of DHD as a descriptor, we examined the niches of the *Pseudoterranova* species and their documented definitive hosts in ecological niche space. We used the R-package ecospat^84^, which is based on a principal components analysis (PCA), with the advantage of removing correlations among the predictor variables, creating the most informative descriptors, based on the original variables.

The PCA-based niche analyses, including Schoener’s D and niche stability, expansion and unfilling, support the concept of the DHD-model. Despite a lower overlap between parasites and some individual hosts, the parasites’ niches are covered completely by the combination of the associated definitive hosts’ niches. Host species with a geographical distribution that is much broader than the parasites’ could result in expansion. This may be the case for *Pusa hispida* and its parasites *Pseudoterranova bulbosa* and *P. decipiens* (*s.s*.), or *Arctocephalus australis* and *P. cattani*. Niche unfilling could indicate that the definitive host spectrum of the parasite might not have been exhaustively recorded yet. There may also be other explanations for niche unfilling, e.g. if the parasites were sampled from a migrating intermediate host. The definitive hosts are still the best option to represent the biotic requirements of the *Pseudoterranova* species, because of the parasites’ low specificity regarding their intermediate hosts.

Under ideal circumstances, data used for habitat suitability models should be sampled with a study design that results in random, unbiased occurrences^85^. Observations should be independent from each other and the data should represent the niche adequately^79^. Therefore, sampling should not be performed in sink populations and the fitness of the sampled population should be assessed^2,86^. However, real-life data does not easily fulfil these requirements. Endoparasites do not allow a direct sampling which is non-invasive to the host species. Considering this, sampling from intermediate hosts is not ideal, because it cannot be assumed that the parasite larva retrieved in a fish will reach maturity. However, since the reproductive stages occur in seals, sufficient sampling from the definitive hosts is not an option. All data occurrences used in this study are independent, since they were sampled in different projects. However, they are not unbiased, since most examined host species are commercially relevant and so are the sampling sites. The demerit of the data available for *Pseudoterranova* spp. is that specimens are rarely identified to species level, even though this is the only way to distinguish larval stages from intermediate hosts. Since only few are molecularly identified, the required number of samples is not available for every species. Occurrence data of the species resident in less accessible areas, as the Southern Ocean, are scarce (e.g. all occurrences of *P. decipiens* E were sampled on the route of the German RV Polarstern). Despite these circumstances, we decided to include all species which use seals as definitive hosts in the study, because it is illustrative to compare the LD- and the DHD-model in species with a range of available occurrences. To account for small sample sizes we used Maxent, which has been reported to perform well in this case^87–90^. We modelled with presence only data, therefore the degree of overprediction cannot be quantified^90^. Galante *et al*.^90^ suggest a combination of model selection and spatial filtering for data-poor species. This approach only works for species whose ecology is well known.

## Conclusion

The life cycle of a parasite is tied to its (several) mandatory hosts. The definitive host is indispensable for the completion of a parasites’ life cycle. Hence, to a certain degree, the distribution of a parasite is determined by the availability of its definitive host. In consequence, the habitat suitability model for a parasite species can be improved by taking into account definitive host distribution. This was done by replacing land distance (used in the LD-models) by definitive host distance (used in the DHD-model). The LD-model shows the potential distribution of *Pseudoterranova* spp., while the DHD-model gives an estimate of its realised distribution.

By including the variable DHD into the model, the Maxent habitat suitability model more represents the observed occurrence of the parasite species. Habitats for boreal and austral species are only modelled in the hemisphere where their hosts actually occur.

## Material and Methods

### Maxent approach

Ecological niche modelling is a correlative approach to assess the present or future habitat suitability of a given geographic area for a certain species. The method links occurrence data to the environmental conditions (most often represented by abiotic descriptors such as climate data) of the sampling area and calculates probabilities of a species’ presence^2,5,91^.

The ENM was performed using Maxent, a machine learning algorithm which assumes that among all models, meeting certain constraints, the model with the highest entropy is most suitable to describe the data^91–93^. Standard settings of Maxent (version 3.3.3) were to calculate linear, quadratic and product features based on Cunze & Tackenberg^94^. In addition, we enhanced the number of iterations in order to ensure convergence. Maxent includes the area under the curve value (AUC-value), which indicates the quality of the model fit. The AUC-value ranges from 0–1 with a value of 0.5 representing a random model^95^. The higher the value the better the specimens are represented by the model. The AUC-values of two models of the same species were compared using the Wilcoxon test.

In order to compile georeferenced reports of *Pseudoterranova* spp., a literature research was conducted (Table S1, Fig. S1). The keyword ‘Pseudoterranova’ was used in the query on Web of Science and Google Scholar. The identification to species level needed to be validated either through molecular methods (such as direct sequencing, PCR-RFLP, enzyme electrophoresis), or by morphological identification of adult stages from definitive hosts. Occurrences referenced as *Pseudoterranova decipiens* (*s.l*.) were not included into the dataset. Spatial autocorrelation was accounted for following the method described in Pearson *et al*.^88^.

The environmental variables used in the model were taken from the global marine environment datasets (GMED). A set of ecologically meaningful factors was chosen to represent the most important factors for the modelling of the parasites and hosts. To avoid overfitting, a correlation analysis of the environmental variables was performed, excluding one variable from a pair of highly intercorrelated variables. In this process, five variables with a Pearson correlation coefficient < 0.51 were chosen: mean sea surface temperature (SST), bottom salinity (Sal), land distance (LD), depth (D) and primary production (PrimProd) with a spatial resolution of 0.083 decimal degrees (5 arc-minutes).

To improve the model consisting solely of abiotic variables (LD-model), definitive host distance (DHD) was included as a biotic predictor, replacing land distance in a new modelling approach (DHD-model). The DHD was created in ESRI ArcGIS by calculating for each gridcell (same spatial resolution as the GMED data) the distance to the nearest gridcell containing at least one definitive host occurrence record. The variable includes the occurrence data of all respective definitive hosts for each *Pseudoterranova* species. The reported definitive host spectrum of the *Pseudoterranova* species was compiled by Mattiucci & Nascetti^96^ (and references therein^33,48,49,97^) and includes 11 different species (*Arctocephalus australis, Cystophora cristata, Halichoerus grypus, Erignathus barbatus, Eumetopias jubatus, Leptonychotes weddelli, Mirounga angustirostris, Otaria flavescens, Phoca vitulina, Pusa hispida, Zalophus californianus*). The host occurrence data (Fig. S2) were retrieved from GBIF.org and Aquamaps.org.

For both models (LD + DHD), the Maxent variable contribution and permutation importance were taken as measures of the relative contributions of the environmental variables to the Maxent models^91^. To review whether the presence of hosts is a positive predictor for parasite presence, the one-variable response curves for the DHD variable are shown. Habitat suitability maps were generated using Esri ArcGIS.

### Ecospat approach

In order to test our hypothesis that the biotic DHD-variable includes more information than LD, we compared the ecological niches of the parasites and associated definitive hosts. We visualized the species’ realized niches based on the PCA_env approach suggested by Broennimann *et al*.^98^ and implemented in the R package “ecospat”^84^. The considered niche space is spanned by the first two principal components of the principal component analysis (PCA) on the five GMED variables used in the abiotic model: mean sea surface temperature (SST), salinity (Sal), land distance (LD), depth (D) and primary production (PrimProd). We calculated Schoener’s D^99,100^, a measure for niche overlap to estimate the extent a parasite’s niche is included in its related definitive host’s niche, niche unfilling, niche expansion and niche stability^101^. This comparison was only shown for *Pseudoterranova bulbosa*, *P. cattani*, *P. decipiens* (s.s.), as only for these species a sufficient amount of occurrence data was available. The niches of *P.bulbosa, P. cattani* and *P. decipiens* (*s.s*.) were analysed with reference to their definitive host niches. Here, niche expansion can be interpreted as the parasite’s niche proportion that does not overlap with the host niche. Niche unfilling is the proportion of the host’s niche that does not overlap with the parasite’s niche^101,102^.

## Supplementary information

Supplementary Information
