# Supplementary material for: Improving species distribution models of zoonotic marine parasites
Source: Sci Rep. 2019 Jul 8;9:9851. doi: 10.1038/s41598-019-46127-6 (PMC6614473; doi:10.1038/s41598-019-46127-6)
Supplement: Supplementary file 1 — Supplementary Information [file 41598_2019_46127_MOESM1_ESM.pdf]

Improving species distribution models of zoonotic marine parasites

Katharina G. Alt[1]*, Judith Kochmann[1], Sven Klimpel[1], Sarah Cunze[1]

[1] Goethe-University, Institute for Ecology, Evolution and Diversity; Senckenberg Biodiversity and Climate Research Centre, Senckenberg Gesellschaft für Naturforschung; Max-von-Laue-Str. 13, D-60438 Frankfurt/Main, Germany

*Corresponding author.

E-Mail: Katharina.Alt@senckenberg.de

ORCID: https://orcid.org/0000-0003-2950-6470

Table S1: *Pseudoterranova* spp. occurrence data.

Molecularly identified, georeferenced *Pseudoterranova* spp. occurrence data with including coordinates, identification method and references.

Pseudoterranova krabbei	*Pseudoterranova decipiens* A				
**Host**	**Location**	**Longitude**	**Latitude**	**Reference**	**Marker**
Haliocherus grypus	NE Atlantic: Faxafloi Bay (Island)	-22.5	64.5	Paggi et al. 2000	Allozym
	NE Atlantic: Faxafloi Bay (Island)	-22.5	64.5	Paggi et al. 1991	Allozym
	NE Atlantic: Frøya Island (Norway)	8.5	63.5	Paggi et al. 1991	Allozym
	NE Atlantic: Frøya Island (Norway)	8.5	63.5	Zhu et al. 2002	rDNA (ITS); SSCP
Phoca vitulina	NE Atlantic: Masfjorden (Norway)	5.5	60.5	Paggi et al. 1991	Allozym
Gadus morhua	NE Atlantic: Møre og Romsdal (Norway)	5.5	62.5	Paggi et al. 1991	Allozym
	NE Atlantic: Faeroe Islands	-6.5	62.5	Paggi et al. 1991	Allozym
	NE Atlantic: FAO 27 (Subarea IV: Iceland, Faroe grounds)	-4.5	62.5	Ferrantelli et al. 2015	rDNA (ITS)
	NE Atlantic: FAO 27 (Subarea V: North Sea)	2.5	57.5	Ferrantelli et al. 2015	rDNA (ITS)
Melanogrammus aeglefinus	NE Atlantic: Faeroe Islands	-6.5	62.5	Paggi et al. 1991	Allozym
Pollachius virens	NE Atlantic: Faeroe Islands	-6.5	62.5	Paggi et al. 1991	Allozym
Hyppoglossoides Platessoides	NE Atlantic: NE Scotland	-2.5	58.5	Paggi et al. 1991	Allozym
Psetta maxima	NE Atlantic: NE Scotland	-2.5	58.5	Paggi et al. 1991	Allozym
Brosme brosme	NE Atlantic: FAO 27 (Subarea IV: Iceland, Faroe grounds)	-4.5	62.5	Ferrantelli et al. 2015	rDNA (ITS)
	NE Atlantic: FAO 27 (Subarea V: North Sea)	2.5	57.5	Ferrantelli et al. 2015	rDNA (ITS)
Chelidonychtis lucernus	NE Atlantic: FAO 27 (Subarea IV: Iceland, Faroe grounds)	-4.5	62.5	Ferrantelli et al. 2015	rDNA (ITS)
	NE Atlantic: FAO 27 (Subarea V: North Sea)	2.5	57.5	Ferrantelli et al. 2015	rDNA (ITS)

Pseudoterranova decipiens (s.s.)	*Pseudoterranova decipiens* B				
**Host**	**Location**	**Longitude**	**Latitude**	**Author**	**Marker**
Halichoerus grypus	NE Atlantic: Faxafloi Bay (Island)	-22.5	64.5	Paggi et al. 2000	Allozym
	NE Atlantic: Faxafloi Bay (Island)	-22.5	64.5	Paggi et al. 1991	Allozym
	NE Atlantic: Frøya Island (Norway)	8.5	63.5	Paggi et al. 1991	Allozym
	NW Atlantic: Anticosti Island (Canada)	-61.5	49.5	Paggi et al. 1991	Allozym
	NW Atlantic: Sable Island (Canada)	-59.5	43.5	Paggi et al. 1991	Allozym
	NW Atlantic: St. Mary's Bay (Canada)	-66.5	44.5	Paggi et al. 1991	Allozym
	NE Atlantic: Polish Baltic coastal Zone	18.5	55.5	Skrzypczak et al. 2014	RFLP, rDNA (ITS)
	NW Atlantic: Anticosti Island (Canada)	-61.5	49.5	Brattey and Davidson 1996	rDNA, RFLP
	NW Atlantic: Isle aux Livres, Quebec (Canada)	-68.5	48.5	Brattey and Davidson 1996	rDNA, RFLP
	NW Atlantic: Sable Island(Canada)	-59.5	43.5	Brattey and Davidson 1996	rDNA, RFLP

	NW Atlantic: Cape Broyle (Newfoundland, Canada)	-52.5	46.5	Brattey and Davidson 1996	rDNA, RFLP
	NW Atlantic: Garden Cove (Newfoundland, Canada)	-54.5	47.5	Brattey and Davidson 1996	rDNA, RFLP
	NW Atlantic: SE Golf of St. Lawrence (Canada)	-61.5	46.5	Brattey and Davidson 1996	rDNA, RFLP
	NW Atlantic: (Newfoundland, Canada)	-54.5	46.5	Brattey and Stenson 1993	Allozym
	NW Atlantic: (Newfoundland, Canada)	-52.5	46.5	Brattey and Stenson 1993	Allozym
	NW Atlantic: (Newscotland, Canada)	-61.5	47.5	Brattey and Stenson 1993	Allozym
	NW Atlantic: (Newscotland, Canada)	-61.5	46.5	Brattey and Stenson 1993	Allozym
	NW Atlantic: (Quebec, Canada)	-61.5	49.5	Brattey and Stenson 1993	Allozym
	NW Atlantic: (Sable Island, Canada)	-59.5	43.5	Brattey and Stenson 1993	Allozym
Phoca vitulina	NW Atlantic: St. Bride´s, Placentia Bay (Newfoundland, Canada)	-54.5	46.5	Paggi et al. 2000	Allozym
	NW Atlantic: Point May (Newfoundland, Canada)	-55.5	46.5	Paggi et al. 2000	Allozym
	NE Atlantic: Matre Masfjorden (Norway)	5.5	60.5	Paggi et al. 2000	Allozym
	NE Atlantic: Tjarno (Sweden)	11.5	58.5	Paggi et al. 1991	Allozym
	NE Atlantic: Husavik (Iceland)	-17.5	66.5	Paggi et al. 1991	Allozym
	NW Atlantic: Sable Island (Canada)	-59.5	43.5	Paggi et al. 1991	Allozym
	NW Atlantic: Point May (Canada)	-55.5	46.5	Paggi et al. 1991	Allozym
	NW Atlantic: St. Brides (Canada)	-54.5	46.5	Paggi et al. 1991	Allozym
	NW Atlantic: St. Marys Bay (Canada)	-66.5	44.5	Paggi et al. 1991	Allozym
	NE Atlantic: Polish Baltic coastal Zone	18.5	55.5	Skrzypczak et al. 2014	RFLP, rDNA (ITS)
	NE Pacific: Rockaway Beach (California, USA)	-122.5	37.5	Nadler et al. 2005	rDNA (LSU, ITS)
	NE Pacific: Richardson Bay (California, USA)	-122.5	37.5	Nadler et al. 2005	rDNA (LSU, ITS)
	NW Atlantic: (Newfoundland, Canada)	-54.5	46.5	Zhu et al. 2002	rDNA (ITS); SSCP
	NW Atlantic: Point May (Canada)	-55.5	46.5	Brattey and Davidson 1996	rDNA, RFLP
	NW Atlantic: St. Marys Bay (Newfoundland, Canada)	-53.5	46.5	Brattey and Davidson 1996	rDNA, RFLP
	NW Atlantic: (Newfoundland, Canada)	-54.5	46.5	Brattey and Stenson 1993	Allozym
	NW Atlantic: (Newfoundland, Canada)	-53.5	46.5	Brattey and Stenson 1993	Allozym
Phoca vitulina richardsii	NE Pacific: Crofton Bay (Canada)	-123.5	48.5	Paggi et al. 1998	Allozym
Cystophora cristata	NW Atlantic: Conception Bay (Canada)	-52.5	47.5	Paggi et al. 1991	Allozym
	NW Atlantic: (Newfoundland, Canada)	-51.5	48.5	Brattey and Stenson 1993	Allozym
	NW Atlantic: (Newfoundland, Canada)	-51.5	49.5	Brattey and Stenson 1993	Allozym
Boreogadus saida	NE Atlantic: Grady Harbour (Canada)	-56.5	53.5	Paggi et al. 1991	Allozym
Brosme brosme	NE Atlantic: Husavik (Iceland)	-17.5	66.5	Paggi et al. 1991	Allozym
	NE Atlantic: FAO 27 (Subarea V: North Sea)	2.5	57.5	Ferrantelli et al. 2015	rDNA (ITS)
Gadus morhua	NE Atlantic: Møre og Romsdal (Norway)	5.5	62.5	Paggi et al. 1991	Allozym

	NE Atlantic: Husavik (Iceland)	-17.5	66.5	Paggi et al. 1991	Allozym
	NW Atlantic: Hermitage Bay (Canada)	-56.5	47.5	Paggi et al. 1991	Allozym
	NW Atlantic: Conception Bay (Canada)	-52.5	47.5	Paggi et al. 1991	Allozym
	NW Atlantic: Passamaquoddy Bay (Canada)	-66.5	45.5	Paggi et al. 1991	Allozym
	NE Atlantic: FAO 27 (Subarea V: North Sea)	2.5	57.5	Ferrantelli et al. 2015	rDNA (ITS)
	NE Atlantic: Eastern Bornholm (Baltic Sea)	15.5	55.5	Mehrdana et al. 2014	rDNA (ITS)
	NE Atlantic: Eastern Bornholm (Baltic Sea), subdivision 25	15.5	54.5	Buchmann and Kania 2012	rDNA (ITS)
	NW Atlantic: South Coast Newfoundland (Canada)	-55.5	45.5	Brattey and Davidson 1996	rDNA, RFLP
	NW Atlantic: Scotian Shelf (Newscotland, Canada)	-61.5	43.5	Brattey and Davidson 1996	rDNA, RFLP
	NW Atlantic: Placentia Bay (Newfoundland, Canada)	-54.5	46.5	Brattey and Davidson 1996	rDNA, RFLP
	NE Atlantic: (Baltic Sea)	16.5	55.5	Zuo et al. 2016	rDNA (ITS)
Gadus ogac	NW Atlantic: Come by Chance (Canada)	-54.5	47.5	Paggi et al. 1991	Allozym
Pollachius virens	NW Atlantic: Passamaquoddy Bay (Canada)	-66.5	45.5	Paggi et al. 1991	Allozym
Myoxocephalus scorpius	NW Atlantic: Point May (Canada)	-55.5	46.5	Paggi et al. 1991	Allozym
	NW Atlantic: NE Coast, Newfoundland (Canada)	-52.5	50.5	Brattey and Davidson 1996	rDNA, RFLP
	NW Atlantic: S Coast, Newfoundland (Canada)	-53.5	45.5	Brattey and Davidson 1996	rDNA, RFLP
Hyppoglossoides Platessoides	NE Atlantic: NE Scotland	-2.5	58.5	Paggi et al. 1991	Allozym
Onchorhynchus gorbuscha	NE Pacific: FAO 67	-153.5	47.5	Bilska-Zając et al. 2016	Genomic DNA
	N Atlantic: N North Sea: Tampen	-1.5	62.5	Klapper et al. 2016	rDNA (ITS)
	N Atlantic: East Greenland	-39.5	63.5	Klapper et al. 2016	rDNA (ITS)
	NE Atlantic: Barents Sea Sea	16.5	74.5	Klapper et al. 2016	rDNA (ITS)
Chelidonychtis lucernus	NE Atlantic: FAO 27 (Subarea V: North Sea)	2.5	57.5	Ferrantelli et al. 2015	rDNA (ITS)
Osmerus eperlanus	NE Atlantic: Cuxhaven (Germany; North Sea)	8.5	53.5	Kuhn et al. 2013	rDNA (ITS)
	NE Atlantic: Elbe Estuary (Germany; North Sea)	8.5	53.5	Zhu et al. 2002	rDNA (ITS); SSCP
Macrourus berglax	N Atlantic: East Greenland Sea	-36.5	64.5	Kellermanns et al. 2007	rDNA (ITS)
Zalophus californianus	NE Pacific: Muir Beach (California, USA)	-122.5	37.5	Nadler et al. 2005	rDNA (LSU, ITS)
	NE Pacific: Monterey (California, USA)	-122.5	36.5	Nadler et al. 2005	rDNA (LSU, ITS)
	NE Pacific: Crofton Bay (Canada)	-123.5	48.5	Paggi et al. 1998	Allozym
Eumetopias jubatus	NE Pacific: Seward (Alaska, USA)	-149.5	59.5	Nadler et al. 2005	rDNA (LSU, ITS)
Mirounga angustirostris	NE Pacific: Santa Cruz (California, USA)	-122.5	36.5	Nadler et al. 2005	rDNA (LSU, ITS)
Enhydra lutris	NE Pacific: Monterey (California, USA)	-122.5	36.5	Nadler et al. 2005	rDNA (LSU, ITS)
Reinhardtius hyppoglossoides	NW Atlantic: Saguenay, Quebec (Canada)	-67.5	49.5	Brattey and Davidson 1996	rDNA, RFLP
Hemiterpterus americanus	NW Atlantic: Grand Manan, New Brunswick (Canada)	-66.5	45.5	Brattey and Davidson 1996	rDNA, RFLP
Hypoglossus hypoglossus	NW Atlantic: Labrador (Canada)	-53.5	53.5	Brattey and Davidson 1996	rDNA, RFLP
	NW Atlantic: Labrador (Canada)	-53.5	52.5	Brattey and Davidson 1996	rDNA, RFLP

Pseudoterranova bulbosa	*Pseudoterranova decipiens* C				
**Host**	**Location**	**Longitude**	**Latitude**	**Author**	**Marker**
Erignathus barbatus	NE Atlantic: Hopen Island (Norway)	25.5	76.5	Paggi et al. 1991	Allozym
	NE Atlantic: Finnmark (Norway)	24.5	71.5	Paggi et al. 1991	Allozym
	NW Atlantic: Makkovic (Canada)	-59.5	55.5	Paggi et al. 1991	Allozym
	N Pacific: Otaru Sea (Japan)	140.5	43.5	Mattiucci et al. 1998	Allozym
	NW Atlantic: Arviat (Hudson Bay)	-94.5	61.5	Karpiej et al. 2014	rDNA (ITS)
	NW Atlantic: Sanikiluaq (Hudson Bay)	-79.5	56.5	Karpiej et al. 2014	rDNA (ITS)
	NW Atlantic: (Newfoundland, Canada)	-55.5	51.5	Zhu et al. 2002	rDNA (ITS); SSCP
	NW Atlantic: Greenspond (Newfoundland, Canada)	-53.5	48.5	Brattey and Davidson 1996	rDNA, RFLP
	NW Atlantic: Noddy Bay (Newfoundland, Canada)	-55.5	51.5	Brattey and Davidson 1996	rDNA, RFLP
	NW Atlantic: Straitsview (Newfoundland, Canada)	-56.5	51.5	Brattey and Davidson 1996	rDNA, RFLP
	NW Atlantic: Black Tickle (Labrador, Canada)	-55.5	53.5	Brattey and Davidson 1996	rDNA, RFLP
	NW Atlantic: Labrador (Newfoundland, Canada)	-55.5	51.5	Brattey and Stenson 1993	Allozym
	NW Atlantic: Labrador (Newfoundland, Canada)	-53.5	53.5	Brattey and Stenson 1993	Allozym
	NW Pacific: Otaru Bay (Japan)	140.5	43.5	Paggi et al. 1998	Allozym
Pusa hispida	NW Atlantic: Arviat (Hudson Bay)	-94.5	61.5	Karpiej et al. 2014	rDNA (ITS)
Hyppoglossoides Platessoides	NE Atlantic: Barents Sea	21.5	73.5	Paggi et al. 1991	Allozym
	NW Atlantic: Labrador (Canada)	-55.5	55.5	Brattey and Davidson 1996	rDNA, RFLP
	NW Atlantic: Labrador (Canada)	-55.5	54.5	Brattey and Davidson 1996	rDNA, RFLP
	NW Atlantic: Labrador (Canada)	-61.5	56.5	Brattey and Davidson 1996	rDNA, RFLP
	NE Atlantic: N Norwegian Waters, Barents Sea	22.5	71.5	Bristow and Berland 1992	morphological, Allozym
	NE Atlantic: N Norwegian Waters, Barents Sea	21.5	71.5	Bristow and Berland 1992	morphological, Allozym
	NE Atlantic: N Norwegian Waters, Barents Sea	18.5	71.5	Bristow and Berland 1992	morphological, Allozym
	NE Atlantic: N Norwegian Waters, Barents Sea	17.5	70.5	Bristow and Berland 1992	morphological, Allozym
	NE Atlantic: N Norwegian Waters, Barents Sea	27.5	72.5	Bristow and Berland 1992	morphological, Allozym
	NE Atlantic: N Norwegian Waters, Barents Sea	29.5	73.5	Bristow and Berland 1992	morphological, Allozym
	NE Atlantic: N Norwegian Waters, Barents Sea	32.5	73.5	Bristow and Berland 1992	morphological, Allozym
	NE Atlantic: N Norwegian Waters, Barents Sea	21.5	73.5	Bristow and Berland 1992	morphological, Allozym
Reinhardtius hyppoglossoides	NW Atlantic: Davis Inlet (Canada)	-60.5	55.5	Paggi et al. 1991	Allozym
	NE Atlantic: Barents Sea	13.5	73.5	Karpiej et al. 2013	rDNA (ITS)
	NW Atlantic: Labrador (Canada)	-53.5	55.5	Brattey and Davidson 1996	rDNA, RFLP
	NW Atlantic: Saguenay, Quebec (Canada)	-67.5	49.5	Brattey and Davidson 1996	rDNA, RFLP
Delphinapterus leucas	NW Atlantic: Kuujjuarapic (Eastern Hudson Bay)	-77.5	55.5	Najda et al. 2015	RFLP, rDNA (ITS)

	NW Atlantic: Kangiqsujuaq (Hudson Street)	-71.5	61.5	Najda et al. 2015	RFLP, rDNA (ITS)
	NW Atlantic: Quaqtaq (Hudson Street)	-69.5	61.5	Najda et al. 2015	RFLP, rDNA (ITS)
Gadus morhua macrocephalus	NW Pacific: Iwanai (Japan)	140.5	42.5	Paggi et al. 1998	Allozym
	NE Pacific: Gulf of Anadyr (Bering Sea)	-179.5	65.5	Paggi et al. 1998	Allozym
Myoxocephalus quadricornis	NE Pacific: Gulf of Anadyr (Bering Sea)	-179.5	65.5	Paggi et al. 1998	Allozym
Hippoglossus hippoglossus	NE Pacific: Gulf of Anadyr (Bering Sea)	-179.5	65.5	Paggi et al. 1998	Allozym

Pseudoterranova azarasi	*Pseudoterranova decipiens* D				
**Host**	**Location**	**Longitude**	**Latitude**	**Author**	**Marker**
Eumetopias jubatus	NW Pacific: Iwanai (Japan)	140.5	42.5	Mattiuci et al. 1998	Allozym
	NW Pacific: Rishiri Island (Japan)	141.5	45.5	Mattiuci et al. 1998	Allozym
	NW Pacific: Iwanai (Japan)	140.5	42.5	Liu et al. 2015	DNA
	NW Pacific: Iwanai (Japan)	140.5	42.5	Zhu et al. 2002	rDNA (ITS); SSCP
	NW Pacific: Rishiri Island (Japan)	141.5	45.5	Paggi et al. 1998	Allozym
	NW Pacific: Otaru Bay (Japan)	140.5	43.5	Paggi et al. 1998	Allozym
	NW Pacific: Iwanai (Japan)	140.5	42.5	Paggi et al. 1998	Allozym
Gadus morhua macrocephalus	NW Pacific: Iwanai (Japan)	140.5	42.5	Mattiuci et al. 1998	Allozym
	NW Pacific	143.5	33.5	Arizono et al. 2011	rDNA (ITS), mtDNA (cox1, NADH)
	NW Pacific: Iwanai (Japan)	140.5	42.5	Paggi et al. 1998	Allozym
	NE Pacific: Gulf of Anadyr (Bering Sea)	-179.5	65.5	Paggi et al. 1998	Allozym
Erignathus barbatus	NW Pacific: Otaru Bay (Japan)	140.5	43.5	Paggi et al. 1998	Allozym
Hippoglossus hippoglossus	NE Pacific: Gulf of Anadyr (Bering Sea)	-179.5	65.5	Paggi et al. 1998	Allozym

Pseudoterranova cattani					
**Host**	**Location**	**Longitude**	**Latitude**	**Author**	**Marker**
Xystreurys rasile	SW Atlantic: Necochea (Argentina)	-58.5	-38.5	Timi et al. 2014	genomic DNA
Paralichthys isosceles	SW Atlantic: Necochea (Argentina)	-58.5	-38.5	Timi et al. 2014	genomic DNA
	SW Atlantic: Patagonian coast (Argentina)	-61.5	-42.5	Hernández-Orts et al. 2013a	mtDNA (cox1)
		-62.5	-42.5	Hernández-Orts et al. 2013a	mtDNA (cox1)
		-61.5	-47.5	Hernández-Orts et al. 2013a	mtDNA (cox1)
		-64.5	-47.5	Hernández-Orts et al. 2013a	mtDNA (cox1)
Paralichthys patagonicus	SW Atlantic: Necochea (Argentina)	-58.5	-38.5	Alarcos et al. 2016	genomic DNA
	SW Atlantic: Quequen (Argentina)	-58.5	-38.5	Timi et al. 2014	rDNA (ITS), mtDNA (cox2)
? (Ceviche)	SE Pacific: Chile (Santiago) (?)	-72.5	-33.5	Weitzel et al. 2015	rDNA (ITS)

Otaria flavescens	SE Pacific: Concepcion (Chile)	-73.5	-36.5	Timi et al. 2014	rDNA (ITS), mtDNA (cox2)
	SW Atlantic: Patagonian coast (Argentina)	-67.5	-49.5	Timi et al. 2014	rDNA (ITS), mtDNA (cox2)
	SW Atlantic: Quequen (Argentina)	-58.5	-38.5	Timi et al. 2014	rDNA (ITS), mtDNA (cox2)
	SW Atlantic: Patagonian coast (Argentina)	-63.5	-40.5	Hernández-Orts et al. 2013b	morphological
		-65.5	-43.5	Hernández-Orts et al. 2013b	morphological
Arctocephalus australis	SW Atlantic: Patagonian coast (Argentina)	-63.5	-40.5	Hernández-Orts et al. 2013b	morphological
		-65.5	-43.5	Hernández-Orts et al. 2013b	morphological
	SW Atlantic: Praia do Mar Grosso, Sao Jose do Norte (Brazil)	-51.5	-31.5	Jacobus et al. 2016	rDNA (ITS), mtDNA (cox2)
Pseudopercis semifasciata	SW Atlantic: Mar del Plata (Argentina)	-58.5	-38.5	Timi et al. 2014	rDNA (ITS), mtDNA (cox2)
		-57.5	-38.5	Timi et al. 2014	rDNA (ITS), mtDNA (cox2)
Achanthistius patachonicus	SW Atlantic: Mar del Plata (Argentina)	-59.5	-39.5	Timi et al. 2014	rDNA (ITS), mtDNA (cox2)
		-60.5	-40.5	Timi et al. 2014	rDNA (ITS), mtDNA (cox2)
	SW Atlantic: Patagonian coast (Argentina)	-61.5	-42.5	Hernández-Orts et al. 2013a	mtDNA (cox1)
		-62.5	-42.5	Hernández-Orts et al. 2013a	mtDNA (cox1)
		-61.5	-47.5	Hernández-Orts et al. 2013a	mtDNA (cox1)
		-64.5	-47.5	Hernández-Orts et al. 2013a	mtDNA (cox1)
Percophis brasiliensis	SW Atlantic: Patagonian coast (Argentina)	-61.5	-42.5	Hernández-Orts et al. 2013a	mtDNA (cox1)
		-62.5	-42.5	Hernández-Orts et al. 2013a	mtDNA (cox1)
		-61.5	-47.5	Hernández-Orts et al. 2013a	mtDNA (cox1)
		-64.5	-47.5	Hernández-Orts et al. 2013a	mtDNA (cox1)
Prionotus nudigula	SW Atlantic: Patagonian coast (Argentina)	-61.5	-42.5	Hernández-Orts et al. 2013a	mtDNA (cox1)
		-62.5	-42.5	Hernández-Orts et al. 2013a	mtDNA (cox1)
		-61.5	-47.5	Hernández-Orts et al. 2013a	mtDNA (cox1)
		-64.5	-47.5	Hernández-Orts et al. 2013a	mtDNA (cox1)
Otaria byronia (= Otaria flavescens)	SE Pacific: Concepcion (Chile)	-73.5	-36.5	Zhu et al. 2002	rDNA (ITS); SSCP
	SE Pacific: Between Cobquecura and Chiloe (Chile)	-72.5	-35.5	George-Nascimento and Urrutia 2000	morphological
	SE Pacific: Between Cobquecura and Chiloe (Chile)	-72.5	-35.5	George-Nascimento and Llanos 1995	morphological, Allozym
Merluccius gayi	SE Pacific: Concepcion (Chile)	-73.5	-36.5	George-Nascimento and Llanos 1995	morphological, Allozym
Genypterus maculatus	SE Pacific: Concepcion (Chile)	-73.5	-36.5	George-Nascimento and Llanos 1995	morphological, Allozym
Paralichthys microps	SE Pacific: Concepcion (Chile)	-73.5	-36.5	George-Nascimento and Llanos 1995	morphological, Allozym

Pseudoterranova decipiens E					
**Host**	**Location**	**Longitude**	**Latitude**	**Author**	**Marker**
Notothenia coriiceps	Southern Ocean: South Shetland Islands (Antarctic)	-59.5	-62.5	Timi et al. 2014	rDNA (ITS), mtDNA (cox2)
Leptonychotes weddellii	Southern Ocean: South Shetland Islands (Antarctic)	-60.5	-62.5	Rengifo-Herrera et al. 2014	mtDNA (cox2)
	Southern Ocean: Weddell Sea (Antarctic)	-51.5	-64.5	Bullini et al. 1997	Allozym
Caenocephalus aceratus	Southern Ocean: South Shetland Islands (Antarctic)	-60.5	-62.5	Zhu et al. 2002	rDNA (ITS); SSCP
Macrourus whitsoni	Southern Ocean: King George and Elephant Island (Antarctic)	-56.5	-61.5	Münster et al. 2016	rDNA (ITS)

Table S2: Environmental descriptors and their contribution to the models.
Variable contribution of the environmental descriptors used in the MaxEnt modelling approaches (LD-model and DHD-model). LD = land distance, DHD = definitve host distance, Salt = salinity, PP = primary production, SST = sea surface temperature (annual mean), depth = bottom depth.

Species	% LD/DHD		% Salt		% PP		% SST		% Depth	
	LD	DHD	LD	DHD	LD	DHD	LD	DHD	LD	DHD
P. azarasi	96.9	57.3	3.0	26.4	0.1	0	0.0	0.3	0.0	16
P. bulbosa	8.8	68.2	3.2	0.1	1.9	7.7	30.8	5.1	55.3	18.9
P. cattani	48.4	75.6	16.2	4.7	16.8	12.6	11.5	1.1	7.1	6
P. decipiens E	2.4	33.2	11.1	20.5	2.3	1.1	83.0	34.3	1.2	10.9
***P. decipiens* (s.s.)**	4.7	53.4	0.1	1.5	21.5	25.2	29.1	8.8	44.6	10.8
P. krabbei	0.5	68.4	0.9	17	0.6	1.1	30.3	1.9	67.7	11.6

Table S3: Model overlap of parasites and definitive hosts.
Schoener's D values calculated for *Pseudoterranova* spp. and their respective hosts. Black outlines and bold letters indicate a host-parasite relationship, green indicates a high, yellow an intermediate and red a low D-value.

	P. azarasi	*P. bulbosa*	*P. cattani*	*P. decipiens (s.s.)*	*P. krabbei*
A. australis	0.029	0.011	**0.179**	0.054	0.010
C. cristata	0.144	0.220	0.194	**0.164**	0.045
E. barbatus	**0.259**	**0.543**	0.058	0.071	0.056
E. jubatus	**0.035**	0.039	0.154	0.121	0.009
H. grypus	0.024	0.009	0.059	**0.450**	**0.011**
M. angustirostris	0.008	0.001	0.146	**0.015**	0.001
O. flavescens	0.116	0.034	**0.328**	0.055	0.056
P. vitulina	0.019	0.016	0.096	**0.383**	**0.007**
P. hispida	0.007	**0.018**	0.004	**0.208**	0.001
Z. californianus	**0.001**	0.000	0.103	**0.008**	0.000

Table S4: Niche comparison of *Pseudoterranova* spp. and their definitive hosts.

Niche comparison of *Pseudoterranova bulbosa*, *P. cattani* and *P. decipiens* (*s.s.*) and their respective hosts. Expansion is the proportion of the parasite's niche that does not overlap with the host niche, stability is the niche overlap and unfilling is the proportion of the host niche that does not overlap with the parasite niche.

	expansion	stability	unfilling
***P. bulbosa* x host**			
P. bulbosa* x *E. barbatus	0.1668111	0.8331889	0.1586211
P. bulbosa* x *P. hispida	0.5422238	0.4577762	0.0000000
***P. cattani* x host**			
P. cattani* x *A. australis	0.5497801	0.4502199	0.0000000
P. cattani* x *O. flavescens	0.0947725	0.9052274	0.1398201
***P. decipiens* (*s.s.*) x host**			
P. decipiens* (*s.s.*) x *C. cristata	0.0596914	0.9403085	0.1584003
P. decipiens* (*s.s.*) x *H. grypus	0.0683955	0.9316044	0.0075531
P. decipiens* (*s.s.*) x *M. angustirostris	0.0523107	0.9476893	0.3131445
P. decipiens* (*s.s.*) x *P. vitulina	0.0840449	0.9159550	0.0520653
P. decipiens* (*s.s.*) x *P. hispida	0.3395640	0.6604360	0.1260158
P. decipiens* (*s.s.*) x *Z. californianus	0.0451382	0.9548617	0.4048093

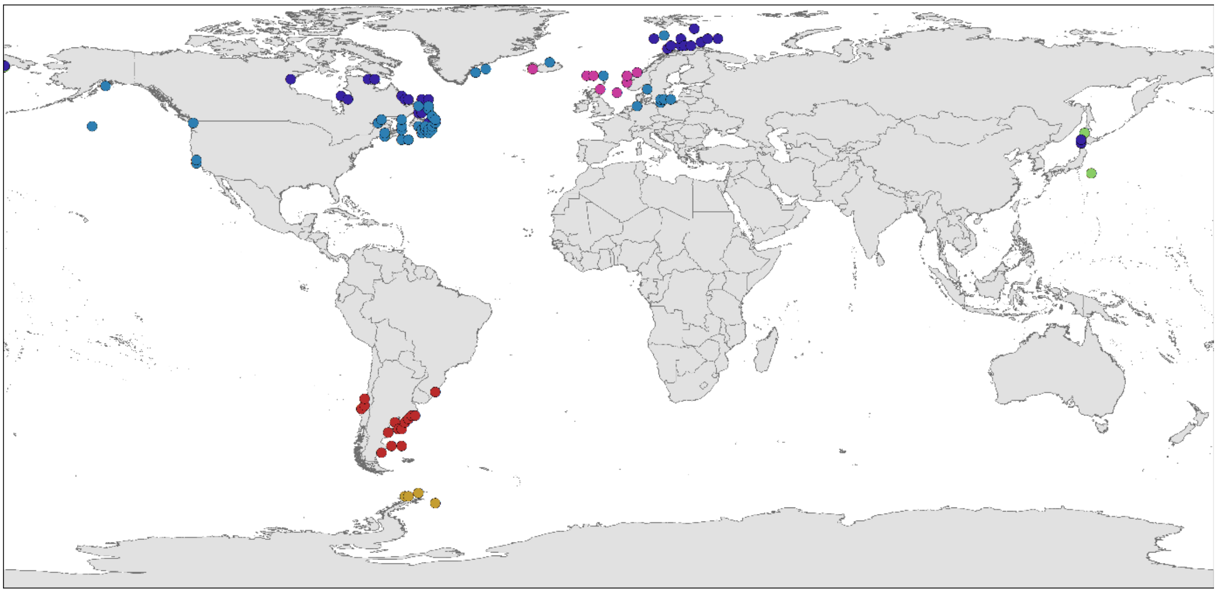

Figure S1: Map of the reported occurrences of *Pseudoterranova* spp.
Occurrences of *Pseudoterranova decipiens* (*s.s.*) (n = 46; light blue), *P. bulbosa* (n = 31, dark blue), *P. cattani* (n = 15, red), *P. krabbei* (n = 8, pink), *P. azarasi* (n = 5, green) and *P. decipiens* E (n = 4, yellow) used for modelling (WGS-84 projection).

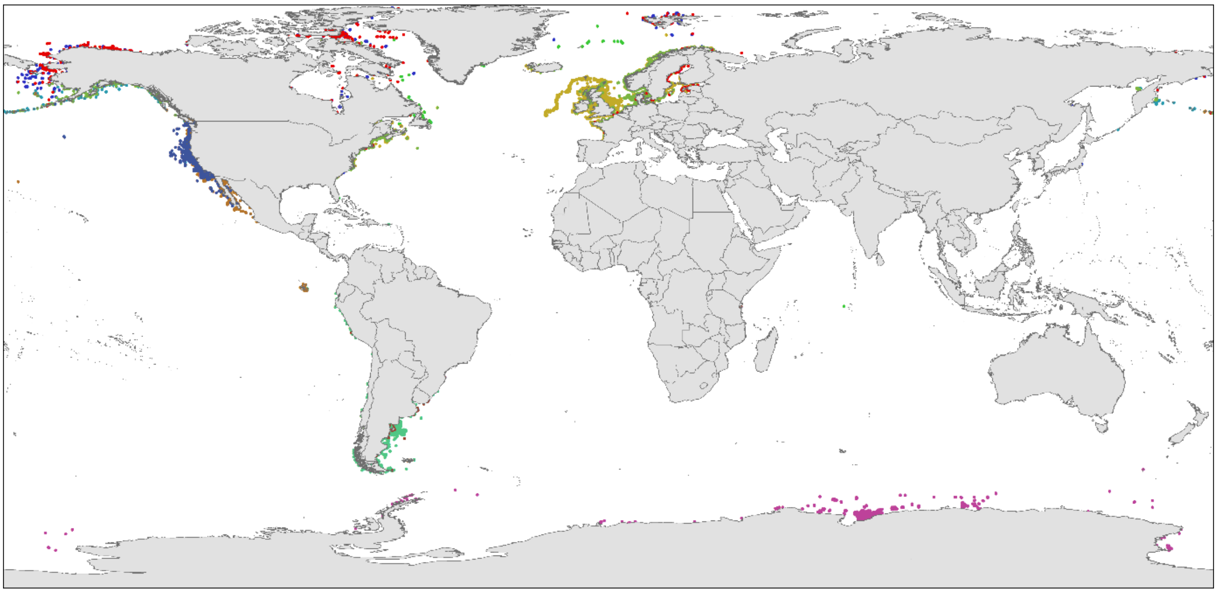

Figure S2: Map of the reported occurrences of the definitive hosts of *Pseudoterranova* spp.
Occurrences of the recorded host species of *Pseudoterranova azarasi* (*Eumetopias jubatus* (n = 919, turquoise), *Zalophus californianus* (n = 4619, ochre), *P. bulbosa* (*Ergnathus barbatus* (n = 573, royal blue), *Pusa hispida* (n = 762, red)), *P. cattani* (*Arctocephalus australis* (n = 42, brown), *Otaria flavescens* (n = 2460, mint green)), *P. decipiens* E (*Leptonychotes weddellii* (n = 5000, pink), *P. decipiens* (*s.s.*) (*Cystophora cristata* (n = 95, green), *Halichoerus grypus* (n = 11317, yellow), *Mirounga angustirostris* (n = 800, blue), *Phoca vitulina* (n = 7368, green), *P. hispida, Z. californianus*) and *P. krabbei* (*H grypus, P. vitulina*) (WGS-84 projection).

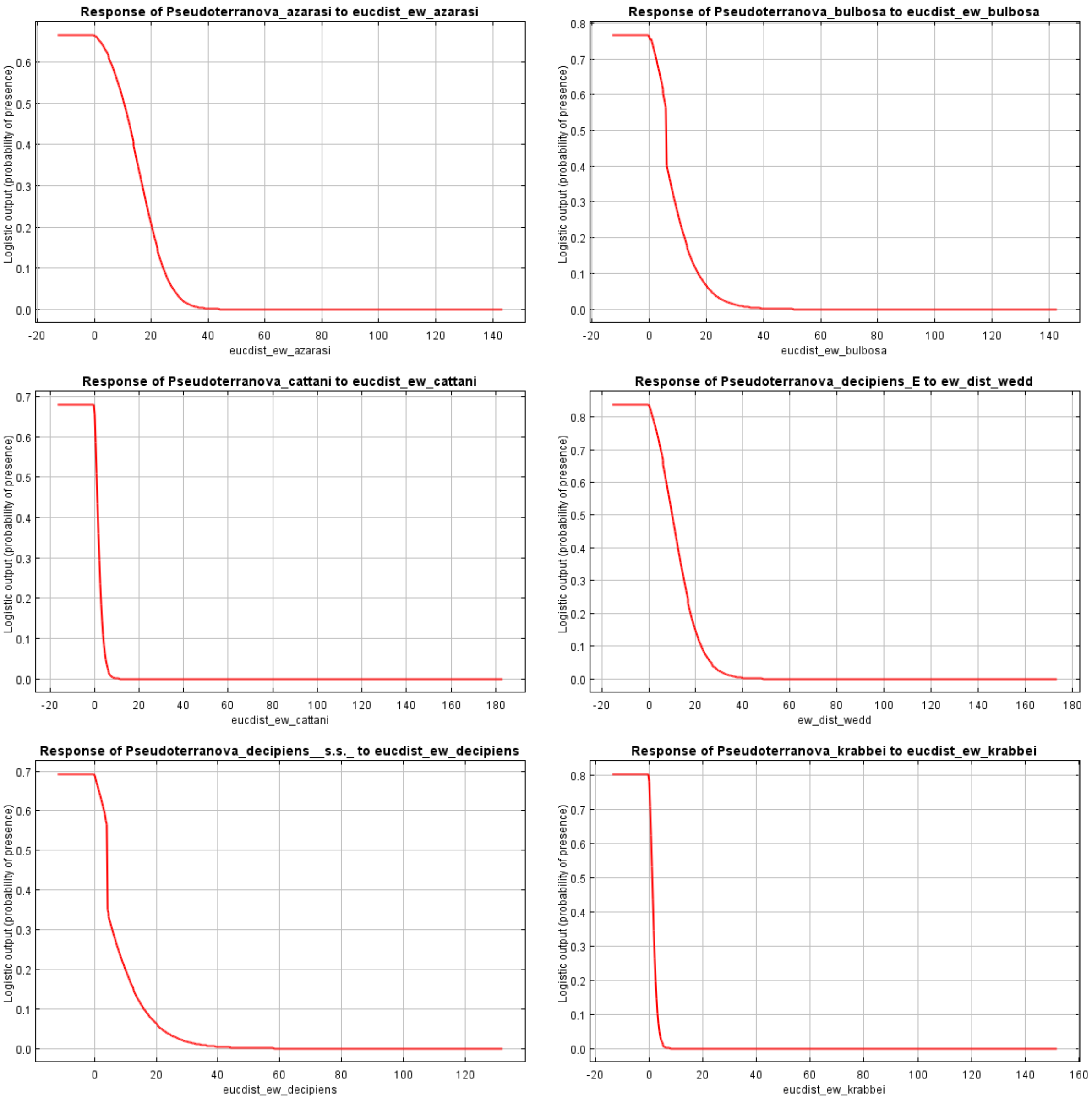

Figure S3: Response curves of definitive host distance.

MaxEnt response curves of DHD for *Pseudoterranova azarasi*, *P. bulbosa*, *P. decipiens* E, *P. decipiens* (*s.s.*) and *P. krabbei*. Probability of presence plotted against the Euclidean distance of the respective definitive host (DHD in decimal degree).

References

1. Paggi, L. *et al. Pseudoterranova decipiens* species A and B (Nematoda, Ascaridoidea): nomenclatural designation, morphological diagnostic characters and genetic markers. *Syst. Parasitol.* **45**, 185–197 (2000).
2. Paggi, L. *et al.* Genetic evidence for three species within *Pseudoterranova decipiens* (Nematoda, Ascaridida, Ascaridoidea) in the North Atlantic and Norwegian and Barents Seas. *Int. J. Parasitol.* **21**, 195–212 (1991).
3. Zhu, X. Q. *et al.* SSCP-based identification of members within the *Pseudoterranova decipiens* complex (Nematoda : Ascaridoidea : Anisakidae) using genetic markers in the internal transcribed spacers of ribosomal DNA. *Parasitology* **124**, 615–623 (2002).
4. Ferrantelli, V. *et al.* Anisakid Nematodes as Possible Markers to Trace Fish Products. *Ital. J. Food Saf.* **4**, 49–53 (2015).
5. Skrzypczak, M., Rokicki, J., Pawliczka, I., Najda, K. & Dzido, J. Anisakids of seals found on the southern coast of Baltic Sea. *Acta Parasitol.* **59**, 165–172 (2014).
6. Brattey, J. & Davidson, W. S. Genetic variation within *Pseudoterranova decipiens* (Nematoda: Ascaridoidea) from Canadian Atlantic marine fishes and seals: characterization by RFLP analysis of genomic DNA. *Can. J. Fish. Aquat. Sci.* **53**, 333–341 (1996).
7. Brattey, J. & Stenson, G. B. Host specificity and abundance of parasitic nematodes (Ascaridoidea) from the stomachs of five phocid species from Newfoundland and Labrador. *Can. J. Zool.* **71**, 2156–2166 (1993).
8. Nadler, S. A. *et al.* Molecular phylogenetics and diagnosis of *Anisakis, Pseudoterranova* and *Contracaecum* from Northern Pacific marine mammals. *J. Parasitol.* **91**, 1413–1429 (2005).
9. Paggi, L. *et al.* Molecular genetics in anisakid nematodes from the Pacific Boreal Region. in *Host Response to International Parasitic Zoonoses* (eds. Ishikura, H., Aikawa, M., Itakura, H. & Kikuchi, K.) 83–107 (Springer Japan, 1998).
10. Mehrdana, F. *et al.* Occurrence of zoonotic nematodes *Pseudoterranova decipiens*, *Contracaecum osculatum* and *Anisakis simplex* in cod (*Gadus morhua*) from the Baltic Sea. *Vet. Parasitol.* **205**, 581–587 (2014).
11. Buchmann, K. & Kania, P. Emerging *Pseudoterranova decipiens* (Krabbe, 1878) problems in Baltic cod, *Gadus morhua* L., associated with grey seal colonization of spawning grounds. *J. Fish Dis.* **35**, 861–866 (2012).
12. Zuo, S. *et al.* Host size-dependent anisakid infection in Baltic cod *Gadus morhua* associated with differential food preferences. *Dis. Aquat. Organ.* **120**, 69–75 (2016).

13. Bilska-Zając, E. *et al.* High prevalence of Anisakidae larvae in marketed frozen fillets of pink salmons (*Oncorhynchus gorbuscha*). *Food Control* **68**, 216–219 (2016).

14. Klapper, R., Kochmann, J., O'Hara, R. B., Karl, H. & Kuhn, T. Parasites as biological tags for stock discrimination of beaked redfish (*Sebastes mentella*): Parasite infra-communities vs. limited resolution of cytochrome markers. *PLOS ONE* **11**, e0153964 (2016).

15. Kuhn, T., Benninghoff, T., Karl, H., Landry, T. & Klimpel, S. Sealworm *Pseudoterranova decipiens* s.s. infection of European smelt *Osmerus eperlanus* in German coastal waters: ecological implications. *Dis. Aquat. Organ.* **102**, 217–224 (2013).

16. Kellermanns, E., Klimpel, S. & Palm, H. W. Molecular identification of ascaridoid nematodes from the deep-sea onion-eye grenadier (*Macrourus berglax*) from the East Greenland Sea. *Deep Sea Res. Part Oceanogr. Res. Pap.* **54**, 2194–2202 (2007).

17. Mattiucci, S. *et al.* Allozyme and morphological identification of shape *Anisakis*, *Contracaecum* and *Pseudoterranova* from Japanese waters (Nematoda, Ascaridoidea). *Syst. Parasitol.* **40**, 81–92 (1998).

18. Karpiej, K., Simard, M., Pufall, E. & Rokicki, J. Anisakids (Nematoda: Anisakidae) from ringed seal, *Pusa hispida*, and bearded seal, *Erignathus barbatus* (Mammalia: Pinnipedia) from Nunavut region. *J. Mar. Biol. Assoc. U. K.* **94**, 1237–1241 (2014).

19. Bristow, G. A. & Berland, B. On the ecology and distribution of *Pseudoterranova decipiens* C (Nematoda: Anisakidae) in an intermediate host, *Hippoglossoides platessoides*, in northern Norwegian waters. *Int. J. Parasitol.* **22**, 203–208 (1992).

20. Karpiej, K., Dzido, J., Rokicki, J. & Kijewska, A. Anisakid nematodes of Greenland halibut *Reinhardtius hippoglossoides* from the Barents Sea. *J. Parasitol.* **99**, 650–654 (2013).

21. Najda, K. *et al.* Anisakidae in beluga whales *Delphinapterus leucas* from Hudson Bay and Hudson Strait. *Dis. Aquat. Organ.* **115**, 9–14 (2015).

22. Liu, S.-S., Liu, G.-H., Zhu, X.-Q. & Weng, Y.-B. The complete mitochondrial genome of *Pseudoterranova azarasi* and comparative analysis with other anisakid nematodes. *Infect. Genet. Evol.* **33**, 293–298 (2015).

23. Arizono, N., Miura, T., Yamada, M., Tegoshi, T. & Onishi, K. Human infection with *Pseudoterranova azarasi* roundworm. *Emerg. Infect. Dis.* **17**, 555–556 (2011).

24. Timi, J. T. *et al.* Molecular identification, morphological characterization and new insights into the ecology of larval *Pseudoterranova cattani* in fishes from the Argentine coast with its differentiation from the Antarctic species, *P. decipiens* sp. E (Nematoda: Anisakidae). *Vet. Parasitol.* **199**, 59–72 (2014).

25. Hernández-Orts, J. S. *et al.* Description, microhabitat selection and infection patterns of sealworm larvae (*Pseudoterranova decipiens* species complex, Nematoda: Ascaridoidea) in fishes from Patagonia, Argentina. *Parasit. Vectors* **6**, 252 (2013).
26. Alarcos, A. J., Pereira, A. N., Taborda, N. L., Luque, J. L. & Timi, J. T. Parasitological evidence of stocks of *Paralichthys isosceles* (Pleuronectiformes: Paralichthyidae) at small and large geographical scales in South American Atlantic coasts. *Fish. Res.* **173**, 221–228 (2016).
27. Weitzel, T. *et al.* Human infections with *Pseudoterranova cattani* nematodes, Chile. *Emerg. Infect. Dis.* **21**, 1874–1875 (2015).
28. Hernández-Orts, J. S. *et al.* Intestinal helminth fauna of the South American sea lion Otaria flavescens and fur seal Arctocephalus australis from northern Patagonia, Argentina. *Journal of Helminthology* (2013). Available at: /core/journals/journal-of-helminthology/article/intestinal-helminth-fauna-of-the-south-american-sea-lion-otaria-flavescens-and-fur-seal-arctocephalus-australis-from-northern-patagonia-argentina/528A883C751D308F4850F8AA5C6393D9. (Accessed: 10th November 2016)
29. Jacobus, K. *et al.* Identification of respiratory and gastrointestinal parasites of three species of pinnipeds (*Arctocephalus australis, Arctocephalus gazella*, and *Otaria flavescens*) in Southern Brazil. *J. Zoo Wildl. Med.* **47**, 132–140 (2016).
30. George-Nascimento, M. & Urrutia, X. *Pseudoterranova cattani* sp. nov. (Ascaridoidea: Anisakidae), un parásito del lobo marino común *Otaria byronia* De Blainville en Chile. *Rev. Chil. Hist. Nat.* **73**, 93–98 (2000).
31. George-Nascimento, M. & Llanos, A. Micro-evolutionary implications of allozymic and morphometric variations in sealworms *Pseudoterranova* sp. (Ascaridoidea: Anisakidae) among sympatric hosts from the Southeastern Pacific Ocean. *Int. J. Parasitol.* **25**, 1163–1171 (1995).
32. Rengifo-Herrera, C. *et al.* Helminth parasites found in faecal samples of phocids from the Antarctic Peninsula. *Polar Biol.* **37**, 685–695 (2014).
33. Bullini, L. *et al.* Genetic and ecological research on anisakid endoparasites of fish and marine mammals in the Antarctic and Arctic-Boreal regions. in *Antarctic Communities: Species, Structure and Survival* (eds. Battaglia, B., Valencia, J. & Walton, D. W. H.) 39–44 (Cambridge University Press, 1997).
34. Münster, J., Kochmann, J., Klimpel, S., Klapper, R. & Kuhn, T. Parasite fauna of Antarctic *Macrourus whitsoni* (Gadiformes: Macrouridae) in comparison with closely related macrourids. *Parasit. Vectors* **9**, 403 (2016).
